# A pyroptosis-related gene signature predicting survival and tumor immune microenvironment in breast cancer and validation

**DOI:** 10.1186/s12885-022-09856-y

**Published:** 2022-09-22

**Authors:** Mingkai Gong, Xiangping Liu, Xian Zhao, Haibo Wang

**Affiliations:** 1 Center of Diagnosis and Treatment of Breast Disease, The Affiliated Hospital of Qingdao University, Qingdao, 266003 People’s Republic of China; 2 Medical Research Center, The Affiliated Hospital of Qingdao University, Qingdao, 266003 People’s Republic of China

**Keywords:** Breast cancer, Bioinformatics, TCGA, pyroptosis-related gene, pyroptosis

## Abstract

**Background:**

Pyroptosis is a newly discovered form of cell programmed necrosis, but its role and mechanism in cancer cells remain unclear. The aim of this study is to systematically analyze the transcriptional sequencing data of breast cancer (BC) to find a pyroptosis-related prognostic marker to predict the survival of BC patients.

**Methods:**

The original RNA sequencing (RNA-seq) expression data and corresponding clinical data of BC were downloaded from The Cancer Genome Atlas (TGCA) database, followed by differential analysis. The pyroptosis-related differentially expressed genes (DE-PRGs) were employed to perform a computational difference algorithm and Cox regression analysis. The least absolute shrinkage and selection operator (LASSO) was utilized to avoid overfitting. A total of 4 pyroptosis-related genes (PRGs) with potential prognostic value were identified, and a risk scoring formula was constructed based on these genes. According to the risk scores, the patients could be classified into high- and low-risk score groups. The potential molecular mechanisms and properties of PRGs were explored by computational biology and verified in Gene Expression Omnibus (GEO) datasets. In addition, the quantitative real time PCR (RT-qPCR) and Human Protein Atlas (HPA) were performed to validate the expression of the key genes.

**Results:**

A PRGs signature, which was an independent prognostic factor, was constructed, and could divide patients into high- and low-risk groups. The results from the prognostic analysis indicated that the survival was significantly poorer in the high-risk group than in the low-risk group both in TCGA and in GEO, indicating that the signature is valuable for survival prediction and personalized immunotherapy of BC patients.

**Conclusions:**

The pyroptosis-related biomarkers were identified for BC prognosis. The findings of this study provide new insights into the development of the efficacy of personalized immunotherapy and accurate cancer treatment options.

## Introduction

Breast cancer is the most common malignancy in females and one of the three most common tumors worldwide [1]. Breast cancer is a heterogeneous subtype of tumor with poor prognosis [2–4]. Due to the development of more effective and superior medical diagnostic and imaging techniques, the mortality rate of BC has been greatly reduced, but the prognosis of patients with BC is still poor [1, 5]. The lack of effective features and diagnostic tools to predict prognosis or long- term survival in patients remains a major obstacle to improve detection and treatment strategies for BC [6].

Pyroptosis distincting from apoptosis, is accompanied by inflammation and immune response, and it is a new form of Gasdermin (GSDM) family-mediated programmed cell death [7, 8].. When bacteria, fungi or parasites invade, immune cells, such as lymphocytes and neutrophils actively kill pathogens through a series of signal transduction. Many pathogens invade and hide in host cells in order to avoid detection by antiseptic substance and phagocytes in body fluids. To eliminate these pathogens, the solution is to clean out them together with the infected cells. Killing infected cells can be done by cell intrinsic mechanisms like necroptosis, apoptosis, and pyroptosis [9]. Gasdermin family member contains gasdermin A, B, C, D, E, and DFNB59 [6, 10]. GasderminD (GSDMD) represents a big gasdermin family, with a new membrane pore forming activity. GSDMD, the substrate of both caspase-11/4/5 and caspase-1, is by far the best researched [11]. Pyroptosis was originally not correctly appreciated for decades because it was similar to apoptosis, and it was condemned as a special type of apoptosis through caspase-1 [12]. Caspase-1 and caspase-11/4/5 induced pyroptosis by cleavage GSDMD, release its gasdermin-N structure domain, the domain structure has the activity of punching holes in the membrane, eventually leading to cell swelling and osmotic lysis. The cells undergo the morphological changes described above [13]. After the demonstration of the GSDMD-mediated pathway, other pyroptosis mechanisms, such as caspase-3/8-mediated pathway and granzyme-mediated pathway, have been clarified by several studies. Chemotherapy can induce caspase-3-mediated cleavage of GSDME, and form N-GSDME terminal, which can cause pyroptosis of tumor cells [14]. Caspase-8 specifically cleaves GSDMC to produce N-GSDMC, and forms pores in cell membrane to induce pyroptosis [15]. Recent studies have proved that GzmB can further activate anti-tumor immune response and inhibit tumor growth by activating caspase-3/GSDME or directly cracking GSDME and inducing pyroptosis [16, 17]. Pyroptosis shows different morphology compared with apoptosis, it is lytic, featuring cell swelling under microscope [18, 19]. Recently, pyroptosis has become a research hotspot in the occurrence and development of tumors. Besides, it is reported to be closely related to gastric cancer, colorectal cancer, hepatocellular carcinoma, breast cancer, and lung cancers [20–25], but its role and mechanism in cancer cells remain unclear.

More and more studies have shown that tumor microenvironment (TME) and tumor stemness are closely related to BC occurrence and development [26, 27], infiltration of numerous inflammatory cells in BC, and the density of CD8+ T cells is highly related to the immune escape of BC [28, 29]. PD-1 and PD-L1 constitute an essential inhibitory mechanism which causes T cell exhaustion in tumor microenvironment. That’s the main reason why PD-L1 has drawn increasing attention of researchers [30–32]. But the underlying mechanisms of pyroptosis in breast cancer microenvironment progression and immune response remain unclear. This study mainly aimed to explore PRGs in BC, and systematically investigate the association between the pyroptosis-related gene signature and immune microenvironment, immune cell infiltration, cancer chemoresistance, cancer stem cells (CSCs). These results supported the feasibility of constructing tumor prognostic risk models using PRGs. At present, few studies have reported the prognostic value of PRGs in BC in recent years.

In this study, the mRNA expression profiles and clinical data of BC patients were first downloaded from TCGA to identify differentially expressed genes (DEGs), especially pyroptosis-related genes. Then, a prognostic signature with these genes was constructed and its reliability was validated in the GEO database. Finally, this gene signature was proved to be able to predict the prognosis of BC and assess the patient’s tumor microenvironment and other states, thereby contributing to clinical treatment.

## Material and methods

### Data collection

**Fig. 1:**
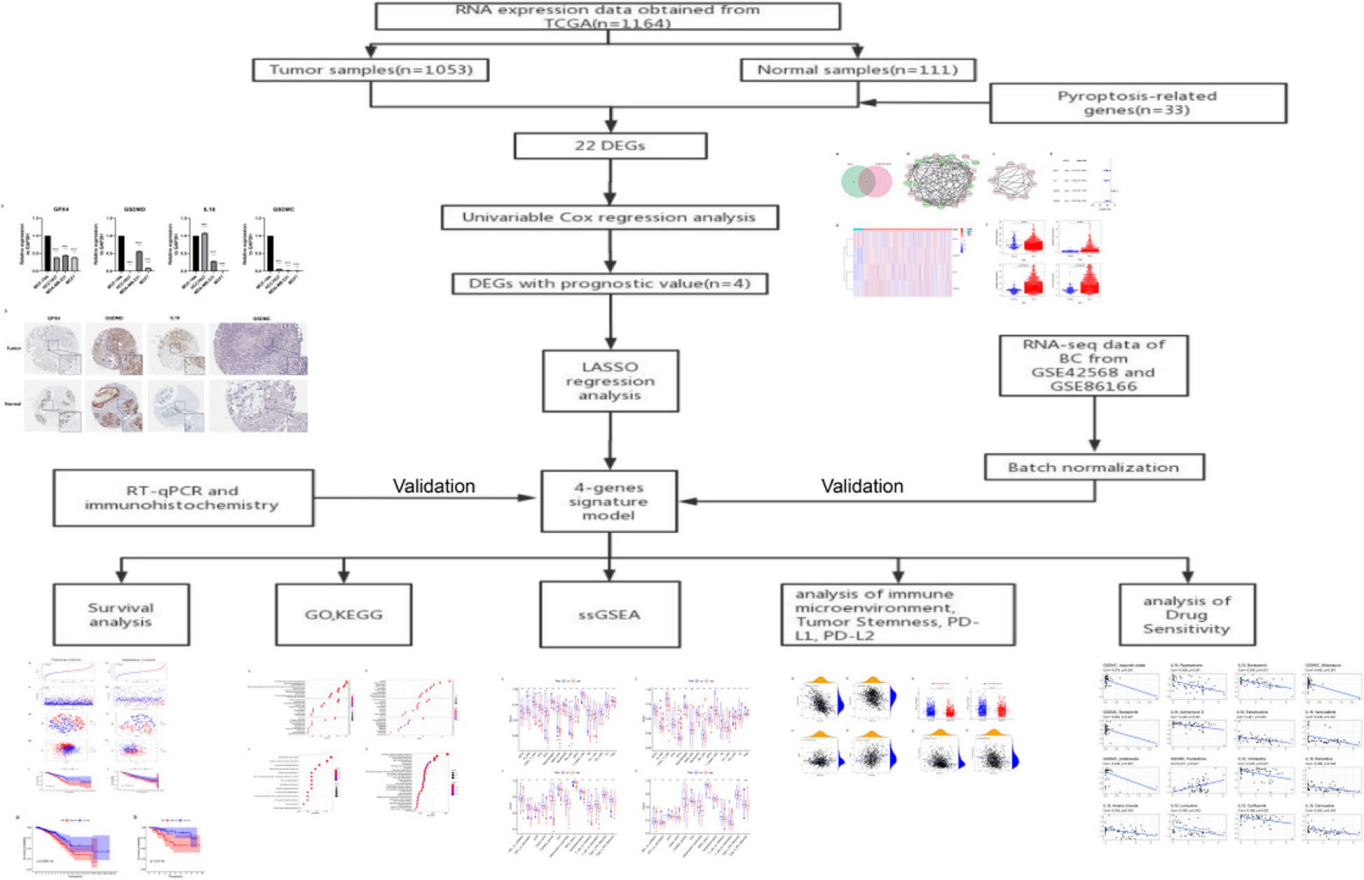
The flow chart of this study

The RNA sequencing (RNA-seq) expression data and clinicopathological information of female breast cancer patients from 1053 breast cancer tissue samples and 111 nontumor tissue samples were downloaded from the TCGA BC dataset (https://portal.gdc.cancer.gov/), and were used as training cohort. Probes were transformed to corresponding Entrez gene names referring to the annotation files. 33 genes associated with pyroptosis were identified from previous literature [33]. In order to get more breast cancer datasets, the GSE42568 and GSE86166 datasets, which were obtained from the gene expression omnibus (GEO: https://www.ncbi.nlm.nih.gov/geo/) database, were used as testing cohort. Batch normalization was applied by using ‘sva’ and ‘limma’ R package [34]. A total of 470 breast cancer samples were obtained. The detailed flow-process diagram of this study is shown in Fig. 1.

### Construction of a pyroptosis-related gene signature

First, the expression level of pyroptosis-related genes was extracted from the total gene expression list. If a gene appeared more than once in the same sample, the ‘limma’ of Bioconductor R package was utilized for averaging operations [35]. Second the limma was utilized to identify DE-PRGs between breast cancer tissue samples and normal breast tissue samples. The false discovery rate (FDR) threshold was set at FDR < 0.05 for DE-PRGs calling. A protein-protein interaction (PPI) network of proteins encoded by DE-PRGs of high-risk epidermal growth factor receptor 2-positive (HER2+) and triple-negative/basal-like molecular subtypes was visualized using String (http://string-db.org). To establish the pyroptosis-related gene risk model, univariate Cox regression analysis was performed on the pyroptosis-related genes. A total of 4 prognostic related differential genes were obtained by the intersection of DE-PRGs and prognostic genes.

To avoid overfitting, the least absolute shrinkage and selection operator (LASSO) was utilized to select variables with high prognostic value [36]. Next, 1000 LASSO iterations were performed for prognostic model construction using the ‘glmnet’ package in R, and their regression coefficients were obtained. Finally, the formula of the risk score was composed as follows, and risk scores were computed: Risk score = ∑ni = ∑Coefi × xi, where xi represents the normalized expression level of target gene i and Coefi represents the regression coefficient. According to the median risk score in TCGA dataset, 1014 patients in the data set were divided into high-risk and low-risk groups after samples with a survival time of zero were removed. The Kaplan-Meier (K-M) plot was used to evaluate survival differences between the high- and low-risk groups. To analyze the distribution differences between different groups, PCA was performed using the ‘prcomp’ function in the STATS package in R. A t-SNE analysis was implemented using the R package Rtsne (https://github.com/jkrijthe/Rtsne).

### Univariate and multivariate cox regression analysis

Univariate cox regression analysis was presented for assessment of the prognostic values of the risk score and clinical features (Age, Stage, T classification, N classification, M classification). Then, multivariate cox regression analysis was used to determine which prognostic factors could independently predict the survival of patients. Adjusted p < 0.05 is considered to be statistically significant using the ‘survival’ package.

### Functional enrichment and pathway analysis

To further investigate the biological processes associated with the pyroptosis-related genes, BC patients were divided into the high- and low-risk groups based on the median risk score in TCGA and testing cohort, Gene Ontology (GO) and Kyoto Encyclopedia of Genes and Genomes (KEGG) pathway enrichment analyses for all selected DEGs between the two risk [37]. Cohorts were performed with the ‘clusterProfiler’ package in BioConductor using |log^2^FC| ≥ 1 and FDR < 0.05 as thresholds in TCGA. Considering the relatively small sample size in testing cohort, the threshold was set as FDR < 0.05.

### Estimation of TME cell infiltration, immuneScore, stromalScore, PD-L1, tumor stemness, drug sensitivity

The scores of 16 tumor-infiltrating immune cells and 13 immune-related functions for samples were determined by single-sample gene-set enrichment analysis (ssGSEA). The ImmuneScore, StromalScore, and ESTIMATEScore were calculated using the ‘ESTIMATE’ package [38, 39]. Correlations between the risk signature and the key immune regulators, PD-L1 and PD-L2 were evaluated. The DNA index is a score based on methylation data and the RNA index is a score based on transcriptome data in TCGA, they can reflect the amount of stem cells [40, 41]. The NCI-60 database and information on 216 FDA-approved chemotherapy drugs were obtained from the CellMiner interface (https://discover.nci.nih.gov/cellminer). Spearman correlation analyses were used to measure the relationship among the risk score, ImmuneScore, StromalScore, PD-L1 and PD-L2 expression, tumor stemness, and drug sensitivity.

### Analysis based on human protein atlas database

The HPA database covers all pathological and gene expression data collected from a large number of studies using different cell lines and tissue types [42]. Immunohistochemistry images in this database were implemented in the present work to examine 4 PRGs levels within diverse tissues along with their localization in cells.

### Cell culture and reagents

Human breast cancer cell lines MCF7, MCF-10A, HCC1937, MDA-MB-231 were provided by Affiliated Hospital of Qingdao University. HCC1937 and MDA-MB-231 ware cultured in DMEM (Invitrogen, USA), MCF7 cells were maintained in RPMI 1640 (Gibco, USA) supplemented with 10% fetal bovine serum (FBS) (Gibco, USA) and 1% penicillin-streptomycin (PS, 100 μg/ml) (Enpromise, Hangzhou, China), MCF-10A was maintained in Medium-F12 (DMEM/F12) (Gibco, USA) supplemented with 5% horse serum (Gibco, USA), 1% penicillin/streptomycin, 0.5 μg/ml hydrocortisone, 100 ng/ml cholera toxin (Sigma, USA), 10 μg/ml insulin (Gibco, USA), and 20 ng/ml recombinant human EGF (Invitrogen, USA). All cells were cultured in a humid environment of 37 °C and 5% CO_2_.

### RNA isolation and quantitative real-time polymerase chain reaction PCR (RT-qPCR)

Total RNA was extracted from cells using TRIzol reagent (Invitrogen, USA). Complementary DNA (cDNA) was synthesized using the total RNA and a PrimeScript RT reagent kit (Takara). TB- Green assays (Takara) were used to perform the RT-qPCR on a Roche LightCycler® 480 instrument. The data was calculated through the 2^-ΔΔCt^ strategy, normalizing with GAPDH. The primer sequences used for qRT-PCR in this study are listed in Table 1.

**Table 1:** Sequences of gene-specific primers used for real-time RT-qPCR

Gene	Forward primer(5′-3′)	Reverse primer(5′-3′)
IL18	GGCTGCTGAACCAGTAGAAGACA	GCTTGCCAAAGTAATCTGATTCCA
GPX4	GAGGCAAGACCGAAGTAAACTAC	CCGAACTGGTTACACGGGAA
GSDMC	CATGCATGGTTTAACCCAAAGG	AACAGGCCAGCAAATCGTGTT
GSDMD	GTGTGTCAACCTGTCTATCAAGG	CATGGCATCGTAGAAGTGGAAG
GAPDH	GAGAAGGCTGGG GCTCATTT	TGATGACCCTTT TGGCTCCC

### Statistical analysis

We used R software (version 4.0.3) to perform all statistical analyses. The Student’s t-test was used to compare gene expression levels between BC samples and non-cancer samples. Heatmaps of the LASSO analysis genes were plotted using the ‘heatmap’ R package. R packages ‘survival’ and ‘survminer’ were used for survival analysis [43, 44]. The OS for the two risk groups was evaluated by Kaplan-Meier (K-M) survival curves and log-rank test. Bioconductor R package ‘GSVA’ was used to compare ssGSEA enrichment scores for immune cells and immune-related pathways between the two groups (i.e. high- or low-risk groups) [45]. Unless otherwise stated, *p < 0.05* was considered statistically significant. P values were showed as: ns not significant; *P < 0.05;**P < 0.01; ***P < 0.001.

## Results

### Identification of prognostic PRGs in the breast TCGA cohort

**Fig. 2:**
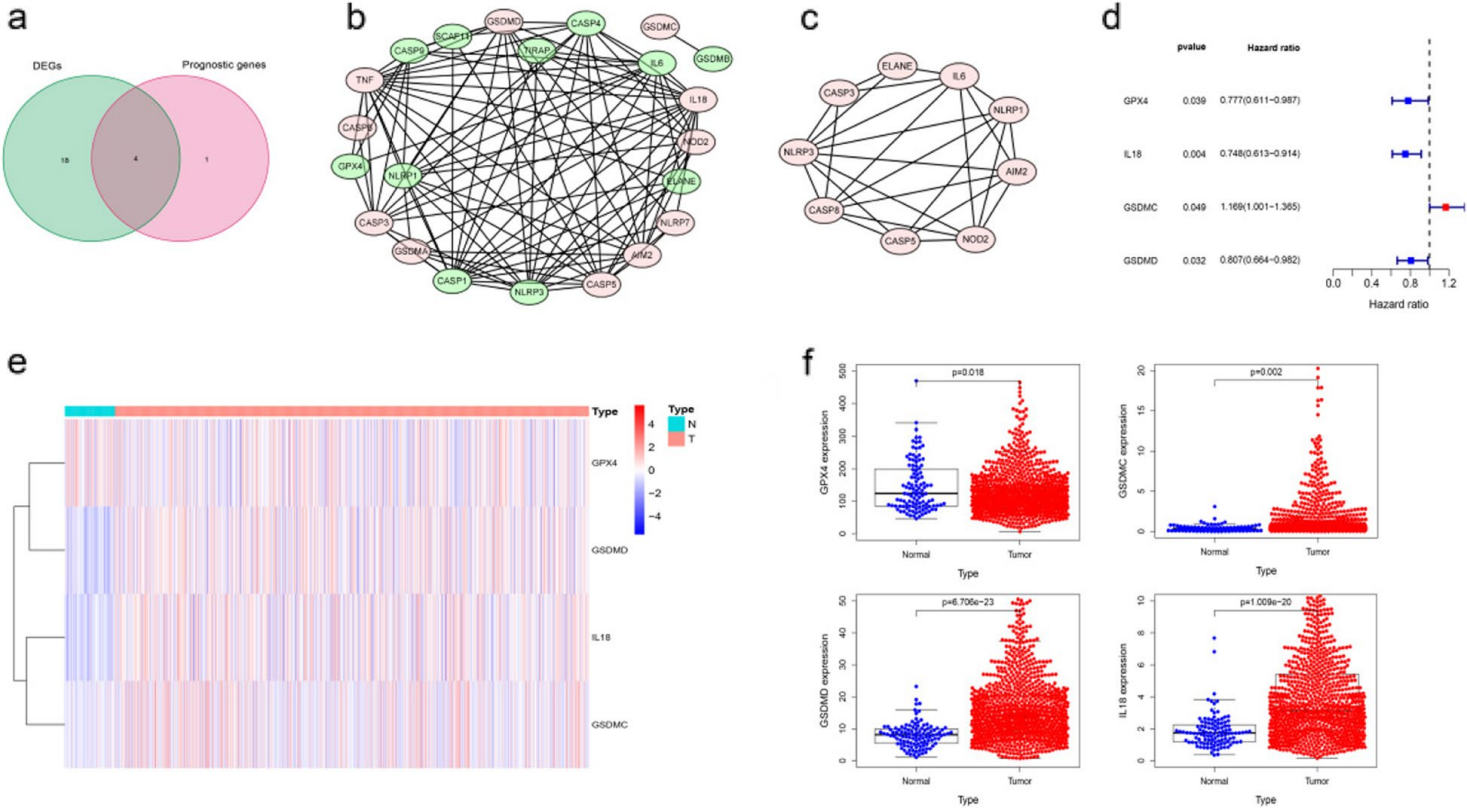
Identification of candidate PRGs in breast cancer. **a** Venn diagram to identify pyroptosis-related DEGs between tumor and adjacent normal tissue that were correlated with OS. **b** Network analysis of internal correlations among DE-PRGs in basal-like molecular subtypes, (**c**) and in HER2+. The red nodes represent the upregulated DE-PRGs, and the green nodes represent the downregulated DE-PRGs. **d** Forest plots showing the results of univariate Cox regression analysis between the expression of 4 candidate PRGs and OS. **e** Heat map showing the expression of 4 candidate PRGs in normal and tumor in breast cancer. **f** The mRNA expression level of 4 PRGs in TCGA

Threshold was set at FDR < 0.05 to compare PRGs expression level between breast cancer and normal tissue. A total of 22 differently expressed genes was obtained. In the univariate Cox regression model, we found 5 genes were associated with a significant OS. Subsequently, a total of 4 intersection genes (*GPX4*, *GSDMD*, *GSDMC*, *IL18*) was selected as hub genes for further analyses (Fig. 2a). The PPI network of HER2+ and basal-like molecular subtypes that indicates tight interplay of pyroptosis-related genes are shown in Fig. 2b and c. Additionally, the prognosis of 4 genes was shown in Fig. 2d and the expression profiles of the 4 genes were showed in a heatmap (Fig. 2e). In order to compare four genes expression level in clinical cases, and to explore the clinical significance of the signature. The mRNA expression level was showed in Fig. 2f, and GPX4 showed a low expression trend, while IL18 showed a high expression.

### Construction of a prognostic PRGs signature

**Fig. 3:**
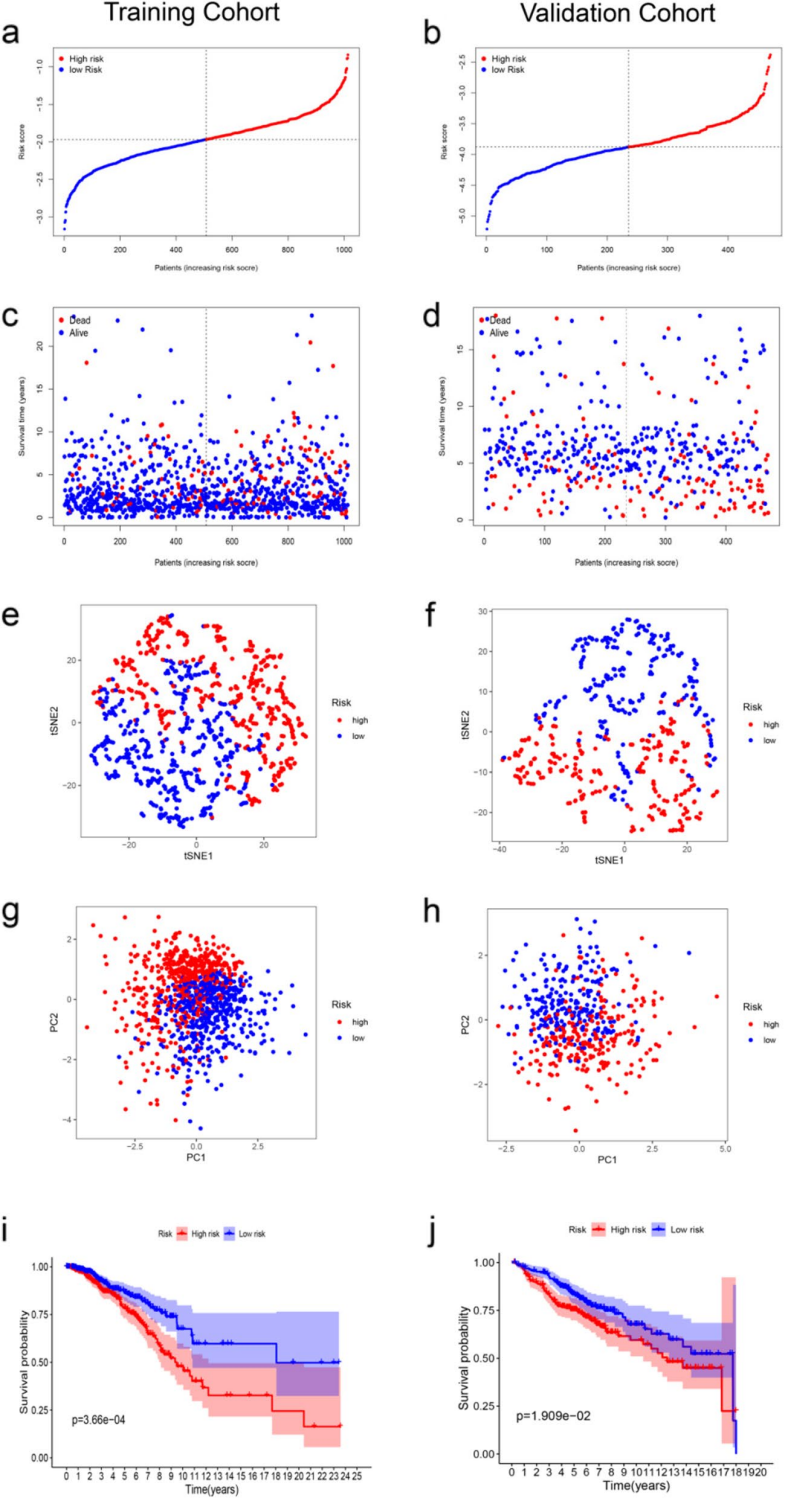
The prognostic performance of the 4 pyroptosis-related gene signature in the training cohort and validation cohort. **a** The distribution of the risk scores in training cohort, (**b**) and in validation cohort. **c** The scatter plots showing whether the samples were alive or not in training cohort, (**d**) and in validation cohort. **e** Two-dimensional projection by a t-SNE analysis in training cohort, (**f**) and in validation cohort. **g** Score plot for the principal component analysis (PCA) in training cohort, (**h**) and in validation cohort. **i** Kaplan-Meier curves for the overall survival of patients in the high- and low-risk groups in training cohort, (**j**) and in validation cohort

According to the result of the LASSO, we ended up with 4 key PRGs which related to prognosis for building the prognostic signature of breast cancer. Figure 3a shows the risk score distribution of patients. Figure 3c shows the survival status of high and low risk group of patients in TCGA database. With the increasing of risk score, the patient’s survival time reduced, on the contrary, the death risk increased. High-risk group of patients than low-risk groups have a greater probability of death incidents. As we can see from t-SNE mappings and PCA (Fig. 3e and g), patients have formed two different clusters. The result of Kaplan-Meier plot shows that the survival rate of high-risk group was obviously lower than low-risk group (Fig. 3i).

### Validation of the prognostic model in the validation cohort

**Fig. 4:**
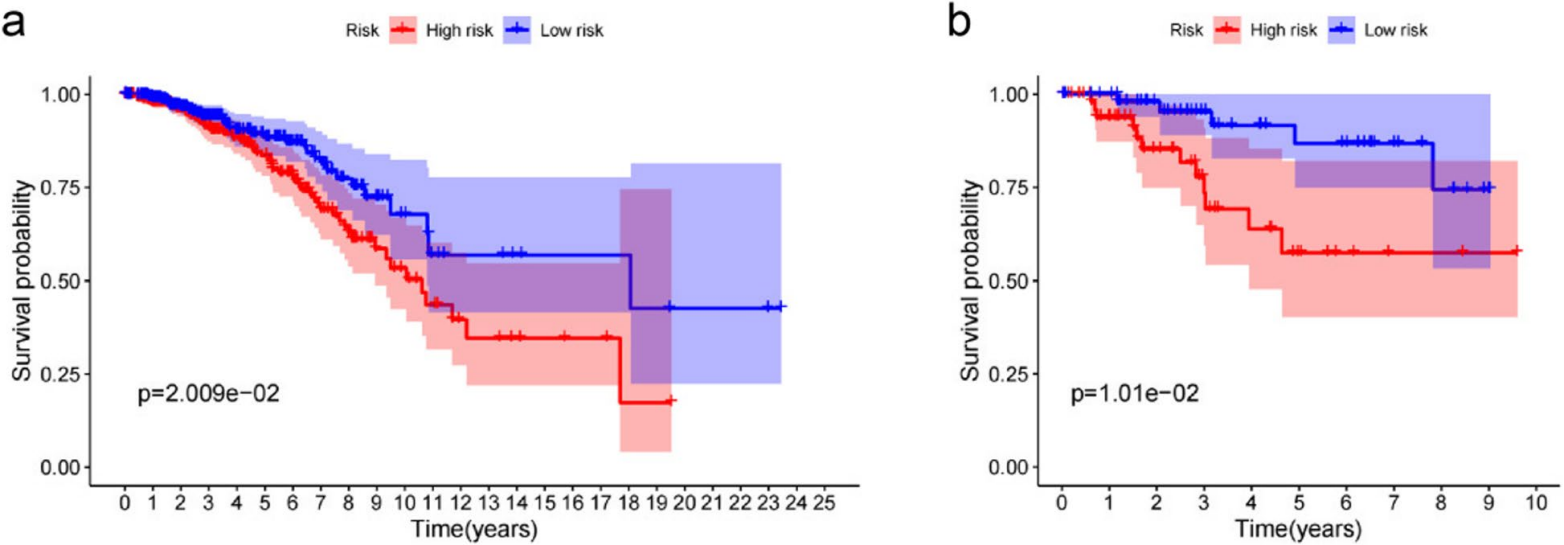
The survival analysis of the pyroptosis-related gene signature in basal-like molecular subtypes and in Luminal. **a** Kaplan-Meier curves of Luminal. **b** Kaplan-Meier curves of basal-like molecular subtypes

To further evaluate the accuracy of the PRGs signature in predicting BC prognosis, it was validated by us using the same methods in the testing cohort. The validation set is segregated into high (*N* = 235) and low (*N* = 235) risk groups, Each patient’s survival outcome, risk status were demonstrated in Fig. 3b, d, f, h. K-M analysis shows that patients in the high-risk group also had a worse prognosis than those in the low-risk group (*P* < 0.05, Fig. 3j). Similarly, the survival rate of high-risk group was significantly lower than that of low-risk group in basal-like molecular subtypes and luminal subtype (*P* < 0.05, Fig. 4a and b).

### Independent prognostic value of the 4-gene signature

**Fig. 5:**
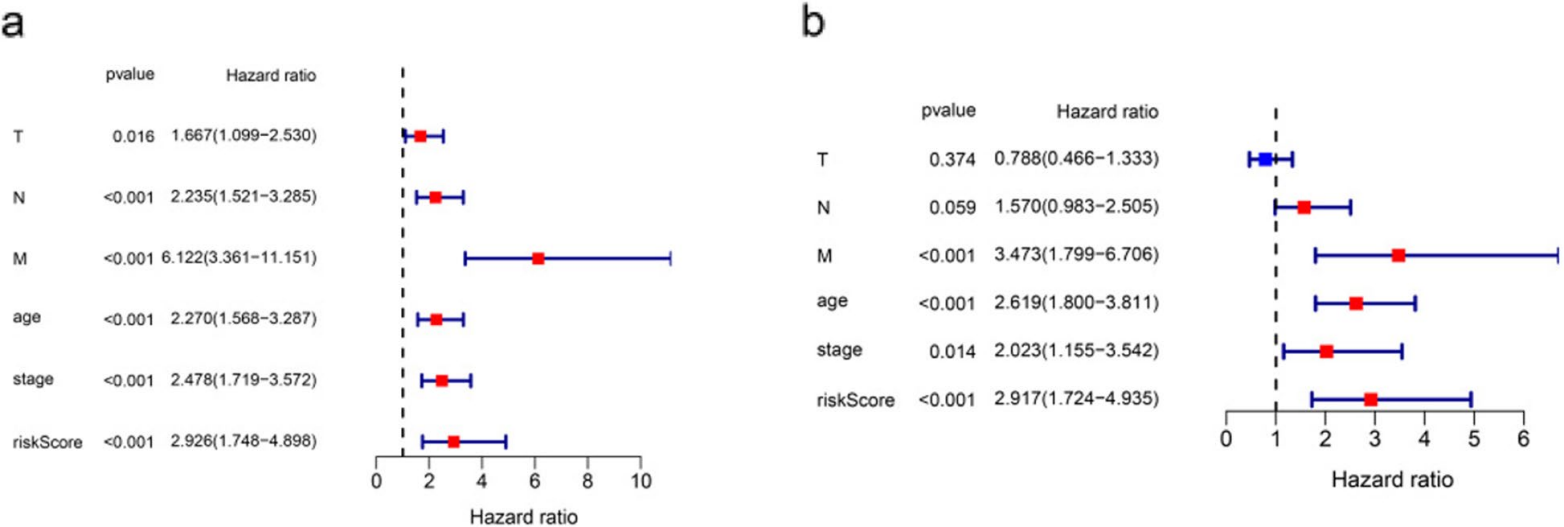
The independency of the pyroptosis-related gene signature for predicting the clinical outcomes for breast cancer in TCGA cohort. **a** Univariate cox regression analysis for assessment of the prognostic values of different clinicopathological characteristics (age, stage, T, N, M) and the risk score. **b** Evaluation of the independency of the risk score and other factors for predicting the prognosis of breast cancer using multivariate cox regression analysis

We observed clinical factors and gene signature prognostic significance through univariate and multivariate regression. Samples have been Chosen with complete clinical information. In 867 cases of patients, according to the age, clinical stage, histological grade and clinical pathologic factors, risk parameters were explored for patients. We have defined these variables indicated significant differences in univariate analysis and stage, age N-classification, M-classification showed significant differences in multivariable analysis. Risk parameters were important prognostic values of *p* < 0.05 **(**Fig. 5a, b and Table 2**)**.

**Table 2:** Univariable and multivariable analyses for each clinical feature

Clinical feature	Number	Univariate Analysis			Multivariate Analysis		
		HR	95% CI	*P* value	HR	95% CI	*P* value
Risk Parameter(high-risk/low-risk)	427/419	2.926	1.748–4.898	<0.001	2.917	1.724–4.935	<0.001
Age(<65/≥65)	626/241	2.270	1.568–3.287	<0.001	2.619	1.800–3.811	<0.001
Stage(I-II/III-IV)	659/208	2.478	1.719–3.572	<0.001	2.023	1.155–3.542	0.014
T(I-II/III-IV)	741/126	1.667	1.099–2.530	0.016	0.788	0.466–1.333	0.374
N(0/1–3)	421/446	2.235	1.521–3.285	<0.001	1.570	0.983–2.505	0.059
M(0/1–3)	851/16	6.121	3.361–11.151	<0.001	3.473	1.799–6.706	<0.001

Abbreviations: T,Tumor;N,Lymph Node;M,Metastasis; HR, hazard ratio; CI, confidential interval

### Functional enrichment analyses

**Fig. 6:**
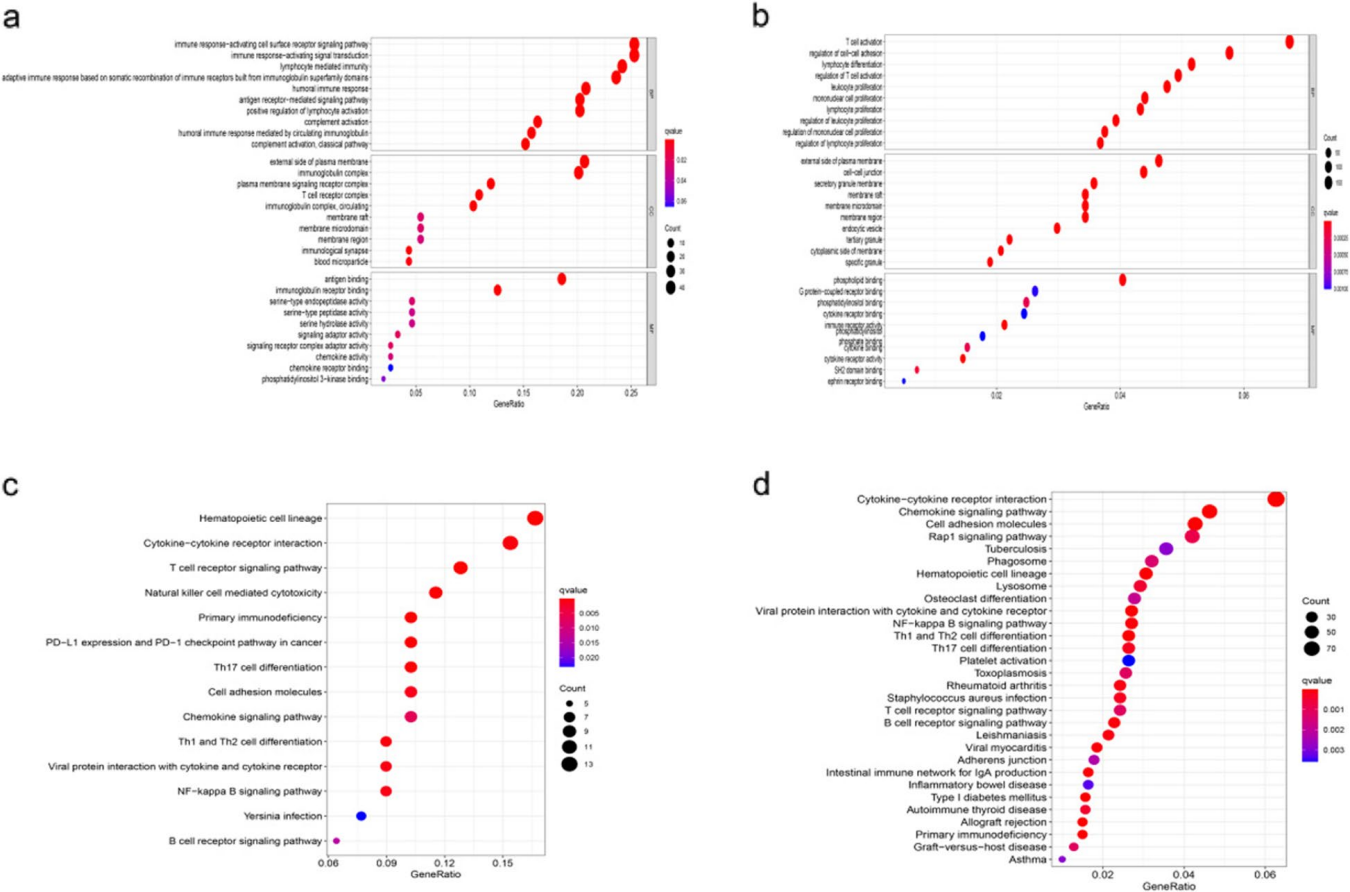
Gene Ontology (GO) and Kyoto Encyclopedia of Genes and Genomes (KEGG) enrichment analysis of 4 biomarkers in The Cancer Genome Atlas (TCGA) and Gene Expression Omnibus (GEO) cohorts. **a** GO enrichment analysis of the 4 PRGs in TCGA cohort. **b** KEGG enrichment analysis of the 4 PRGs in TCGA cohort. **c** GO enrichment analysis of the 4 PRGs in the GEO cohort. **d** KEGG enrichment analysis of the 4 PRGs in the GEO cohort

**Fig. 7:**
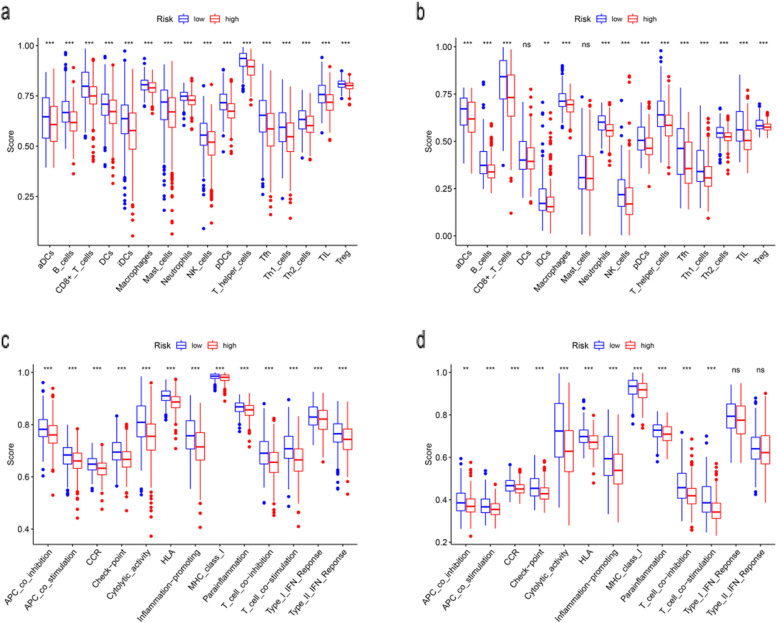
Comparison of the ssGSEA scores for immune cells and immune pathways. **a**, **c** Comparison of the enrichment scores of 16 types of immune cells and 13 immune-related biological processes between low- (blue box) and high-risk (red box) group in the TCGA cohort. **b**, **d** Comparison of the tumour immunity between low- (blue box) and high-risk (red box) group in the GEO cohort

GO and KEGG functional enrichment analyses were performed on risk-related DEGs to investigate the potential functions. The result of GO analyse indicated that the DEGs equally concentrated in membrane raft, membrane microdomain, membrane region and external side of plasma membrane (Fig. 6a, b). And KEGG functional enrichment analysis suggested that the DEGs were mainly related to Cytokine-cytokine receptor interaction, Hematopoietic cell lineage, etc. both in TCGA and GEO database (Fig. 6c, d). To further explore the relationship between BC prognosis and immune status, we quantified immune cell infiltration score and immune-related function using ssGSEA. The correlations between ssGSEA scores and different risk groups showed that the scores of iDCs, aDCs, CD8 + Tcells, T helper cells, NK cells, Macrophages, Th2-cells, Treg were higher in the low-risk group (Fig. 7a, b). Meanwhile, APC costimulation, cytolytic_activity, inflammation−promoting, parainflammation, etc. were significantly different between the low- and high-risk groups in both TCGA and GEO database **(**Fig. 7c, d**)**.

### Associations with immunity, tumor stemness, and drug sensitivity

**Fig. 8:**
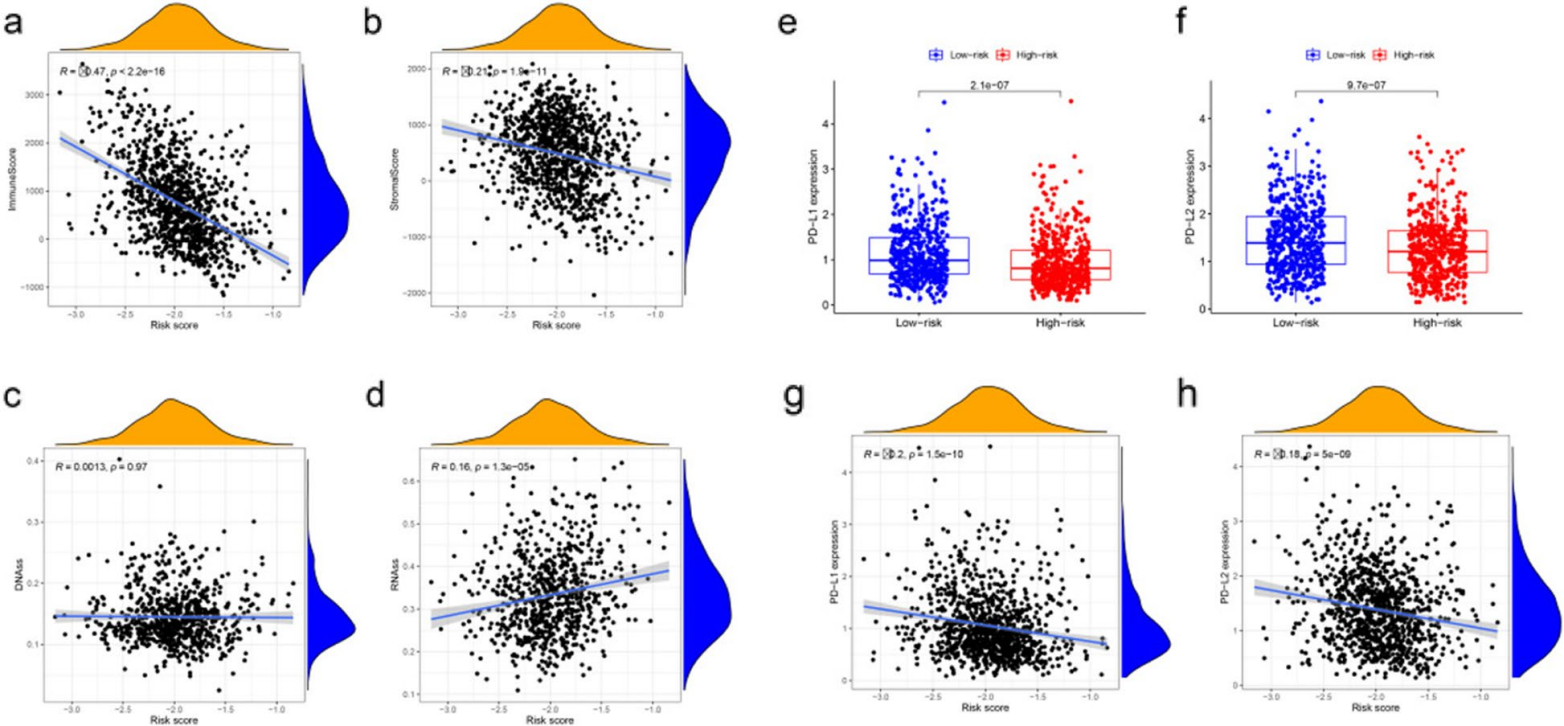
Potential role of risk signature in BC immune status, tumor stemness. **a** immune scores (**b**) stromal scores (**c**) DNAss (**d**) RNAss (**e**) Expression levels of genes PD-L1 among two risk subgroups in BC patients. **f** and PD-L2 (**g**) Correlation analysis between risk score, PD-L1 (h) and PD-L2

**Fig. 9:**
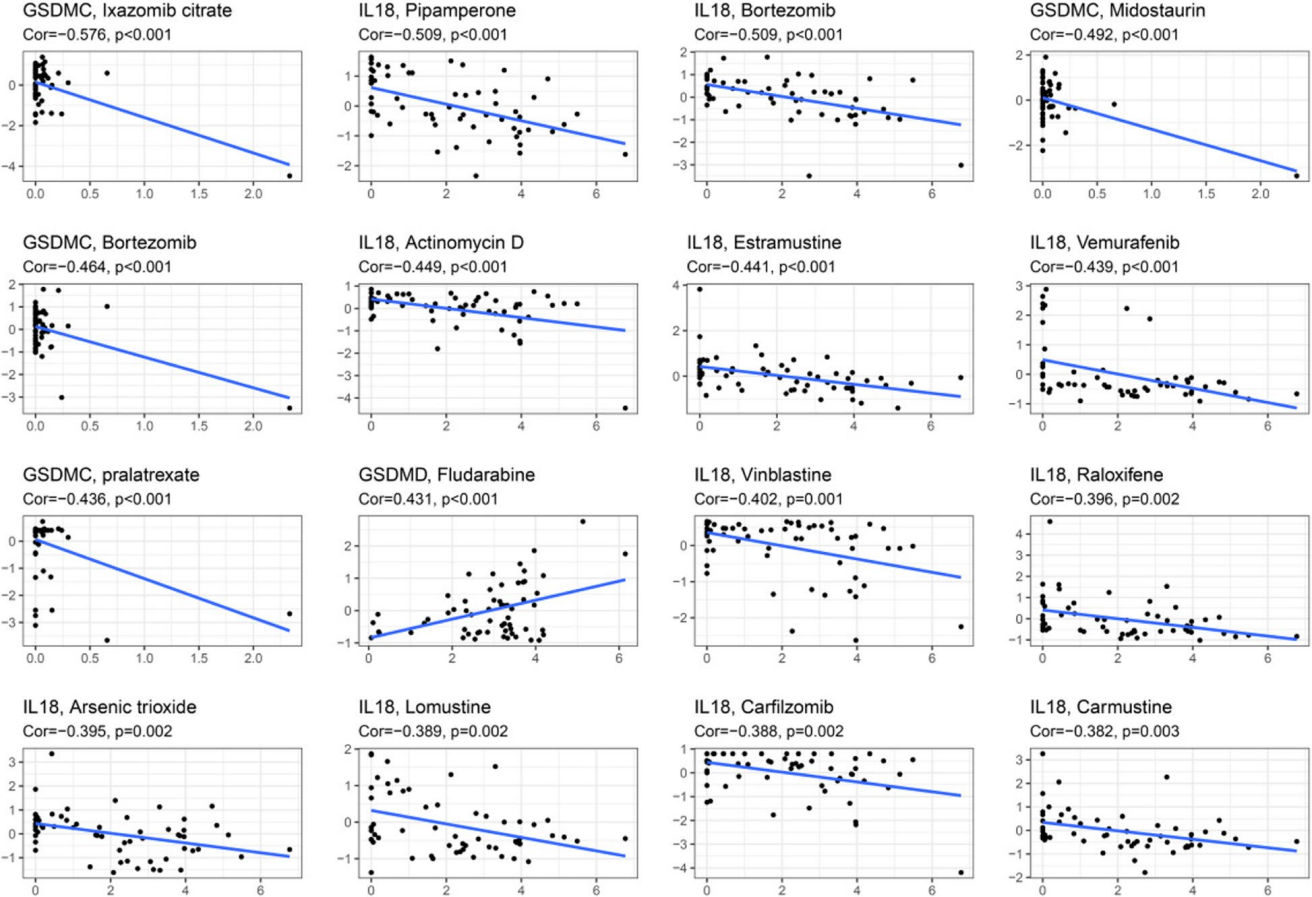
Scatter plots of top 16 classes of associations between hub pyroptosis genes and drug sensitivity

The constructed risk signature was significantly negatively correlated with the immune and stromal scores (Fig. 8a, b). Furthermore, there are no significant correlation between PyroptosisScore and DNAss (Fig. 8c), but positively correlated with RNAss (Fig. 8d). When studying the relationship with immune checkpoints, considering the role of PD-L1 (also known as CD274) and PD-L2 (also known as PDCD1LG2) in immune microenvironment and immune escape, we analyzed the difference in the expression of these two proteins between high and low risk groups. The results showed that the protein expression levels of PD-L1 and PD-L2 in the high risk group were significantly lower than those in the low risk group (Fig. 8e, f), and the protein expression level was negatively correlated with the risk score (Fig. 8g, h). As shown in Table 3, the PRGs are resistant to most drugs (*p* < 0.05). For example, Paclitaxel, Vinorelbine, Gemcitabine and Epirubicin are commonly used drugs to treat breast cancer, the expression levels of IL18 were negatively associated with tumor cell sensitivity to Paclitaxel, Vinorelbine and Epirubicin. In contrast, sensitivity to the chemotherapy drug Gemcitabine was positively associated with the expression levels of GSDMD in tumor cells (Fig. 9).

**Table 3:** Sensitivity correlation analysis between the independent prognostic genes and drugs based on the CellMiner Database

Gene	Drug	cor	*p *value
GSDMC	Ixazomib citrate	−0.57595479	1.47E-06
IL18	Pipamperone	−0.509024906	3.28E-05
IL18	Bortezomib	−0.508793631	3.31E-05
GSDMC	Midostaurin	−0.491860473	6.57E-05
GSDMC	Bortezomib	−0.463557491	0.000191399
IL18	Actinomycin D	−0.448998313	0.00032019
IL18	Estramustine	−0.440708258	0.000424876
IL18	Vemurafenib	−0.439369013	0.000444445
GSDMC	pralatrexate	−0.436058325	0.000496385
GSDMD	Fludarabine	0.431453794	0.000577795
IL18	Vinblastine	−0.402432989	0.001434584
IL18	Raloxifene	−0.396260817	0.001723
IL18	Arsenic trioxide	−0.394839745	0.001796347
IL18	Lomustine	−0.389369145	0.002105486
IL18	Carfilzomib	−0.387952286	0.00219293
IL18	Carmustine	−0.382263852	0.002577528
IL18	Depsipeptide	−0.380139576	0.002735875
GSDMC	Vismodegib	−0.379539246	0.002782165
GSDMD	Cladribine	0.379224295	0.002806728
GSDMC	Gefitinib	0.378968948	0.002826784
IL18	Ixazomib citrate	−0.377554717	0.002940186
IL18	Sulfatinib	−0.373232429	0.003312239
GSDMC	Vincristine	−0.368981618	0.003718274
IL18	Paclitaxel	−0.368430241	0.003774044
IL18	VINORELBINE	−0.360484258	0.00466406
GPX4	Selumetinib	−0.359505	0.004785642
IL18	Mithramycin	−0.359091919	0.004837761
IL18	Dabrafenib	−0.354169816	0.005498363
IL18	Homoharringtonine	−0.349851754	0.006141874
IL18	Vincristine	−0.341962731	0.007489547
GPX4	ARRY-162	−0.329858532	0.010058815
IL18	Vinorelbine	−0.328589792	0.010367904
IL18	Doxorubicin	−0.323741261	0.011626145
IL18	ETHINYL ESTRADIOL	−0.321734287	0.012184365
IL18	ARSENIC TRIOXIDE	−0.321556026	0.012235046
GPX4	Cobimetinib (isomer 1)	−0.319111208	0.012948702
GSDMD	Vinorelbine	−0.317517609	0.013432945
IL18	Irofulven	0.314947083	0.014246885
IL18	Epirubicin	−0.314263548	0.014470323
GPX4	Digoxin	0.312582561	0.015032651
GSDMD	Depsipeptide	−0.308463009	0.016490554
IL18	Teniposide	−0.308415202	0.016508159
IL18	Tamoxifen	−0.305102479	0.017767832
GPX4	Floxuridine	0.304346956	0.018066337
GSDMD	Ixazomib citrate	0.302574225	0.018783572
GSDMC	Dacomitinib	0.297738692	0.020864437
IL18	Tegafur	−0.296503111	0.021426482
IL18	Crizotinib	−0.296205911	0.021563573
GSDMD	Eribulin mesilate	−0.290597914	0.024293174
IL18	Afatinib	0.288715478	0.02527245
GSDMD	Nelarabine	0.283957854	0.027896746
IL18	Ixabepilone	−0.282683139	0.028637519
IL18	Encorafenib	−0.282663266	0.028649197
GSDMD	6-THIOGUANINE	0.281349635	0.029430039
IL18	Dacomitinib	0.281147048	0.029552033
IL18	Abiraterone	−0.278419627	0.031236075
GSDMD	Clofarabine	0.278163601	0.031398193
GSDMD	Floxuridine	0.278052059	0.031469043
GSDMD	Vismodegib	0.277730639	0.03167395
GSDMD	Gemcitabine	0.276118299	0.032718712
GPX4	LEE-011	−0.275011314	0.033452526
GPX4	Trametinib	−0.273321502	0.034599099
IL18	Erlotinib	0.273073537	0.034770066
IL18	Etoposide	−0.271955429	0.035549733
IL18	Nilotinib	−0.270877836	0.036314838
GPX4	Denileukin Diftitox Ontak	−0.27071664	0.036430455
GSDMD	Actinomycin D	−0.270591993	0.036520068
GPX4	Temsirolimus	0.270096569	0.036878052
GSDMC	Idarubicin	−0.26925671	0.037491565
GSDMD	Mithramycin	−0.268870062	0.03777684
GPX4	LDK-378	−0.268764945	0.037854707
IL18	Eribulin mesilate	−0.267440211	0.038847471
GSDMC	Carmustine	−0.266647795	0.039451539
GSDMC	DAUNORUBICIN	−0.265426515	0.040397727
GSDMC	Erlotinib	0.263596821	0.041850285
GPX4	Ibrutinib	0.26305371	0.042289643
GSDMD	Cytarabine	0.263023079	0.042314535
GSDMD	Vinblastine	−0.261927998	0.04321241
GSDMC	Bisacodyl, active ingredient of Viraplex	0.258236502	0.046355329
GSDMC	Pazopanib	−0.257997618	0.046565006
GSDMD	Neratinib	−0.25748124	0.047020905
GPX4	Vinorelbine	−0.257398217	0.047094544
GSDMD	Paclitaxel	−0.25630594	0.048072193

### Expression levels of key genes in the clinical samples and RT-qPCR

**Fig. 10:**
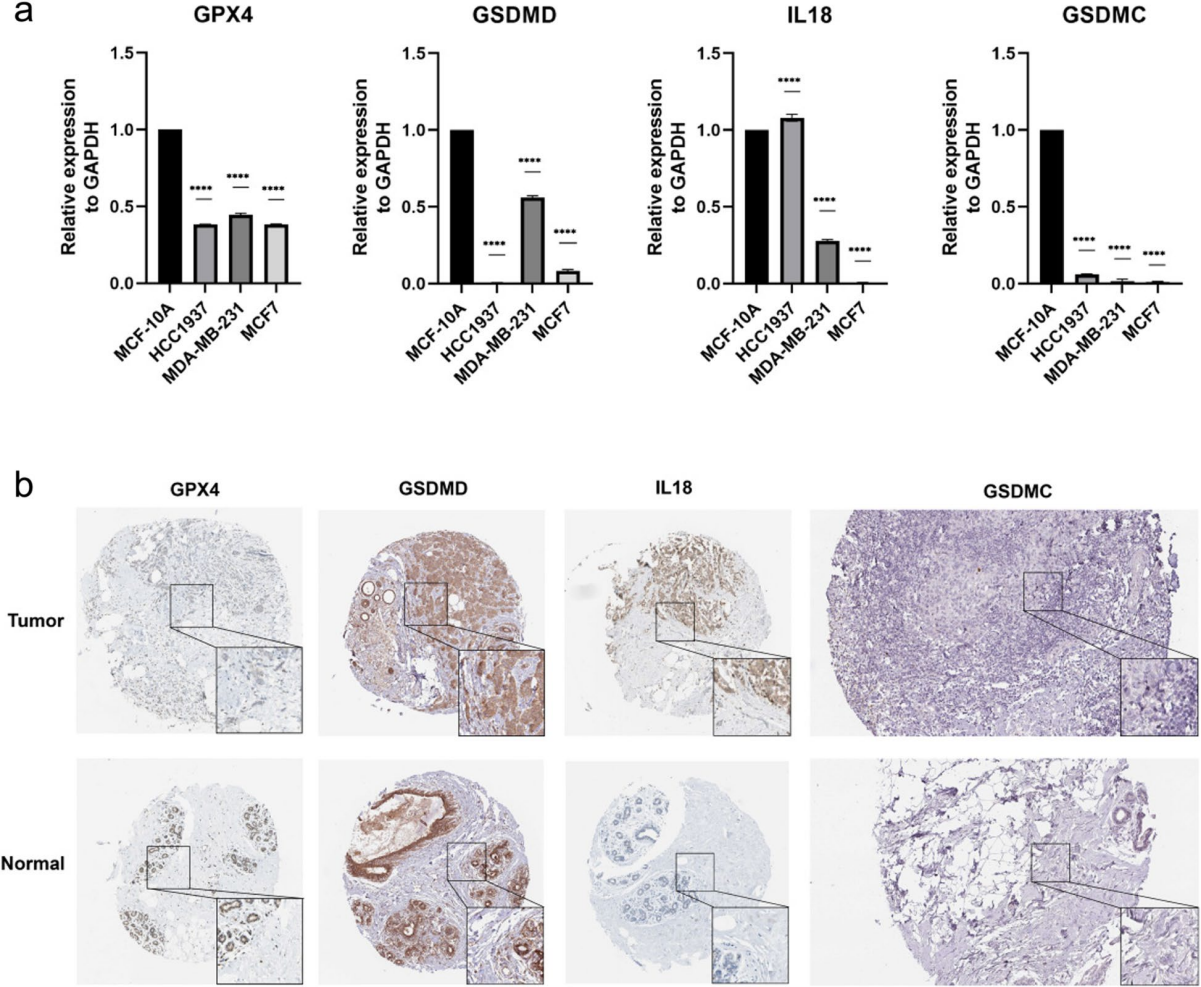
The mRNA and Immunohistochemistry of PRGs expression. **a** Differences in mRNA levels of PRGs between normal and various tumor cells. **b** BC tumor and normal breast tissue images are shown for the PRGs-coded proteins

To confirm the bioinformatics prediction, we detected the expression of the four PRGs by RT-qPCR. It showed a low mRNA expression trend of *GPX4*, *GSDMD* and *GSDMC* in all three types of breast cancer cells, while the mRNA expression of *IL18* varied (Fig. 10a). The immunohistochemistry results were studied by using the HPA database in normal breast tissue and tumor tissue to explore the clinical significance of the signature. The results showed the expression and distribution of *GPX4*, *GSDMD*, *GSDMC*, *IL18* in breast cancer and normal tissues (Fig. 10b).

## Discussion

Breast cancer is one of the most common tumors in women. At present, the treatment of breast cancer includes surgery, chemotherapy and radiotherapy. Although the cure rate has been greatly increased, so far, there is still no effective method to accurately predict the prognosis of patients with BC. Previous studies have found many tumor molecular markers of BC. The detection and targeted treatment of estrogen receptor, progesterone receptor and human epidermal growth factor receptor 2 have been widely used in clinical practice [46]. But the lack of accurate prognostic biomarkers are still the main problem with improve clinical outcomes in patients with breast cancer. In recent years, with the correct understanding of pyroptosis, the latest research has found that pyroptosis plays an important role in the occurrence and development of tumors [47, 48]. Therefore, the study of biomarkers related to pyroptosis is expected to treat breast cancer more accurately.

In this study, 4 pyroptosis-related genes were found differently expressed in breast cancer and related to prognosis according to TCGA. So, we firstly establish a pyroptosis-related genes signature in the context of BC before evaluating its prognostic value and clinical significance. Then, patients with BC were classified according to the expression of pyroptosis-related genes. In order to obtain more samples and verify the feasibility of signature, two sets of GEO data were downloaded, normalized and integrated. Our gene signature was found to be able to predict prognosis in BC patients with high accuracy in training and testing cohorts. In addition, the risk score was identified as an excellent independent prognostic factor characterized by good sensitivity and specificity.

Then we further explore the relationship between pyroptosis and BC, The high-risk group was also rich in biological processes related to malignant progression, and there were significant differences between the two risk model subgroups of BC patients in both the training and testing cohorts. In the high-risk group, almost all immune cell infiltration and immune function were suppressed. Given the critical roles of these immune cells in stimulating anti-tumor immunity [49], it is reasonable to conclude that anti-tumor immunity was significantly reduced in the high-risk group of BC patients. In addition, the ESTIMATE algorithm showed that the stromal and immune cell scores were both inversely associated with risk scores, confirming poor immune cell infiltration in the high-risk subgroup. Triple negative breast cancer (TNBC) has a poor prognosis and high mortality compared to other breast cancers [50], so intensive efforts have been made to develop treatments targeting TNBC. Cancer immunotherapy targeting PD-L1 has improved outcomes for TNBC [49], and shown efficacy in other several cancers [51]. PD-L1 and PD-L2 are key regulators of immune responses [52, 53]. This study also verified that PD-L1 and PD-L2 were significantly different in the two risk subgroups, and both were negatively correlated with risk score. The levels of nearly all immune checkpoints were significantly lower in the high-risk subgroup than in the low-risk subgroup, indicating that the immune response was dramatically altered in this group. Comprehensive analysis of immune cells, immune function, immune-related markers and PRGs confirmed the important role of pyroptosis in immune regulation in TME landscape. CSCs are considered as the major cause to tumor initiation, recurrence, metastasis, and drug resistance, driving poor clinical outcomes in patients [54]. In this study, the risk signature was positively correlated with the stem cell score, confirming that our newly constructed gene signature was a risk factor for BC. Some researchers have been studying the role of pyroptosis-related genes in the development of cancer. And the downregulation of GSDMD was found to attenuate tumor proliferation via the intrinsic mitochondrial apoptotic pathway and inhibition of EGFR/Akt signaling and predicted a good prognosis in non-small cell lung cancer [24]. However, it has also been proved that down-regulation of GSDMD promotes gastric cancer proliferation by regulating cell cycle-related proteins and over-expression of GSDMC is a prognostic factor for predicting a poor outcome in lung adenocarcinoma [20, 55]. So, more experiments should be done to confirm our findings.

Despite the prognostic value of the risk signature, there are several limitations in this study. First of all, this was a retrospective analysis, thus, prospective studies are needed to confirm the results. Secondly, there is a lack of experimental analysis to validate the results of bioinformatics analyses. In the future, more functional studies are needed to understand pyroptosis-related genes and their role in BC development.

## Conclusion

In conclusion, 4 pyroptosis-related genes were found associated with BC prognosis in this study. The signature was proved to be independently associated with OS in TCGC cohort and GEO validation cohort. More significantly, it was found extremely valuable in functional analysis, tumor microenvironment, and drug sensitivity, providing insight for predicting the prognosis of BC. But the specific potential mechanism between pyroptosis-related genes and tumor immunity is still unclear and deserves further study. Our work will help shed light on the role of pyroptosis in tumgenesis, particularly in the areas of immune response, tumor microenvironment and drug resistance, which are crucial for the development of personalized cancer therapies.

## Data Availability

The public datasets used in our work can be found on https://portal.gdc.cancer.gov/ and https://www.ncbi.nlm.nih.gov/geo/. Immunohistochemical datasets are available on https://www.proteinatlas.org/.

## Abbreviations

BC: breast cacner
TNBC: Triple negative breast cancer
TGCA: The Cancer Genome Atlas
GEO: Gene Expression Omnibus
HPA: Human Protein Atlas
PRGs: pyroptosis-related genes
DE-PRGs: pyroptosis-related differentially expressed genes
LASSO: least absolute shrinkage and selection operator
TME: tumor microenvironment
OS: overall survival
GSDM: Gasdermin
CSCs: Cancer stem cells
RNA-seq: RNA sequencing
FDR: false discovery rate
K-M: Kaplan-Meier
GO: Gene Ontology
KEGG: Kyoto Encyclopedia of Genes and Genomes
ssGSEA: single-sample gene-set enrichment analysis
FBS: fetal bovine serum
cDNA: Complementary DNA
